# Oxygenation effect of interventional lung assist in a lavage model of acute lung injury: a prospective experimental study

**DOI:** 10.1186/cc4889

**Published:** 2006-04-07

**Authors:** Günther Zick, Inéz Frerichs, Dirk Schädler, Gunnar Schmitz, Sven Pulletz, Erol Cavus, Felix Wachtler, Jens Scholz, Norbert Weiler

**Affiliations:** 1 Department of Anesthesiology and Intensive Care Medicine, University Hospital Schleswig-Holstein, Campus Kiel, Germany

## Abstract

**Introduction:**

The aim of the study was to test the hypothesis that a pumpless arteriovenous extracorporeal membrane oxygenator (interventional lung assist (ILA)) does not significantly improve oxygenation in a lavage model of acute lung injury.

**Methods:**

The study was designed as a prospective experimental study. The experiments were performed on seven pigs (48–60 kg body weight). The pigs were anesthetized and mechanically ventilated. Both femoral arteries and one femoral vein were cannulated and connected with ILA. Acute lung injury was induced by repeated bronchoalveolar lavage until the arterial partial pressure of O_2 _was lower than 100 Torr for at least 30 minutes during ventilation with 100% O_2_.

**Results:**

ILA was applied with different blood flow rates through either one or both femoral arteries. Measurements were repeated at different degrees of pulmonary gas exchange impairment with the pulmonary venous admixture ranging from 35.0% to 70.6%. The mean (± standard deviation) blood flow through ILA was 15.5 (± 3.9)% and 21.7 (± 4.9)% of cardiac output with one and both arteries open, respectively. ILA significantly increased the arterial partial pressure of O_2 _from 64 (± 13) Torr to 71 (± 14) Torr and 74 (± 17) Torr with blood flow through one and both femoral arteries, respectively. O_2 _delivery through ILA increased with extracorporeal shunt flow (36 (± 14) ml O_2_/min versus 47 (± 17) ml O_2_/min) and reduced arterialization of the inlet blood. Pulmonary artery pressures were significantly reduced when ILA was in operation.

**Conclusion:**

Oxygenation is increased by ILA in severe lung injury. This effect is significant but small. The results indicate that the ILA use may not be justified if the improvement of oxygenation is the primary therapy goal.

## Introduction

The mortality of patients with acute respiratory distress syndrome (ARDS) has remained high at about 30–50% despite all efforts in research and treatment [1]. Different strategies of mechanical ventilation focusing on the avoidance of ventilator-induced lung injury [2,3] and on the recruitment of diseased lung areas [4,5] are considered in the management of respiratory failure. Additional approaches applied are prone positioning [6], high-frequency oscillatory ventilation [7,8] and extracorporeal membrane oxygenation [9-11].

The venovenous and venoarterial application of extracorporeal membrane oxygenation is often associated with coagulation and bleeding complications, with activation of the inflammatory cascade, with damage of red blood cells and with technical problems [12-14]. These adverse effects mainly result from the use of long tubings, heat exchangers and external pumps. To reduce the incidence of such complications, pumpless arteriovenous systems for extracorporeal gas exchange have recently been developed.

These pumpless arteriovenous systems have low priming volumes, short tubings and small foreign surface areas, and they therefore exhibit less adverse effects than classical extracorporeal membrane oxygenation. The pumpless lung assist devices are only effective in hemodynamically stable patients, however, because the natural arteriovenous blood pressure gradient determines the flow through the oxygenator. An animal experimental study has even shown that continuous hemodynamic support was necessary with pumpless extracorporeal lung assist [15].

Although the pumpless extracorporeal lung assist has been shown to improve the CO_2 _removal [16-19], its oxygenation effect is difficult to assess. This is mainly because the blood entering the oxygenator is already of arterial origin and the amount of oxygen that can be added by the oxygenator is limited. The lower the oxygen saturation of the inlet blood, however, the greater the effect expected. Another limitation of the extracorporeal lung assist is the fact that only a small fraction of the cardiac output passes through the oxygenator and only this blood is supplied with oxygen. On the return of blood into the systemic circulation, the oxygen saturation decreases considerably due to the mixture with venous blood. A significant oxygenation effect of the extracorporeal lung assist device can only be expected when sufficient flow through the oxygenator is secured.

Since the oxygenation effect of the pumpless extracorporeal lung assist has not extensively been studied until now and only the effective CO_2 _removal has been well described, partly under the conditions of normal lung function [20], the primary aim of our study was to test the hypothesis that a pumpless arteriovenous extracorporeal membrane oxygenator does not significantly improve oxygenation in a lavage model of severe acute lung injury. We expect that our study may provide new information on the possible use of the extracorporeal lung assist to improve oxygen supply in ARDS patients.

## Materials and methods

The study was approved by the university committee for animal care and adhered to the guidelines on animal experimentation. The experiments were performed on seven domestic pigs (Deutsches Landschwein, Institute of Animal Breeding and Husbandry, Christian-Albrechts-University, Kiel, Germany) with a body weight of 48–60 kg. The animals were sedated with azaperon (8 mg/kg) in combination with atropine (0.1 mg/kg). Anesthesia was induced with ketamine (5 mg/kg) and, after cannulation of an ear vein, sufentanil (0.2 μg/kg) and propofol (1 mg/kg) were added. The pigs were intubated and ventilated with a Siemens servo 900 C ventilator (Siemens-Elema, Solna, Sweden) with an inspired oxygen fraction (FiO_2_) of 1.0, a tidal volume of 9 ml/kg body weight at a positive end-expiratory pressure of 5 cmH_2_O and a respiratory rate of 20 breaths/minute. During preparation and instrumentation the ventilator settings were set to attain normal levels of arterial partial pressure of oxygen (PaO_2_) and of arterial partial pressure of carbon dioxide (PaCO_2_). Anesthesia was maintained with propofol (6–8 mg/kg per hour) and sufentanil (10 μg/kg per hour).

A catheter was introduced into the carotid artery, allowing continuous analysis of PaO_2 _and PaCO_2 _(Paratrend 7+; Diametrics Medical Inc, High Wycombe, UK) and arterial pressure measurement. This access was also used for arterial blood sampling. The samples were processed by a blood gas analyzer (ABL System 615; Radiometer Medical Inc., Copenhagen, Denmark), which was also applied for the measurement of hemoglobin concentration. A pulmonary artery catheter was inserted through the internal jugular vein to provide central venous, pulmonary artery and capillary wedge pressures, as well as continuous cardiac output (Baxter Healthcare, Irvine, CA, USA). Mixed venous blood samples were drawn through this line. The heart rate, the partial pressure of CO_2 _in respired gas, the airway pressures, and the pulmonary artery, arterial and central venous pressures were monitored using the S/5 anesthesia monitoring system (Datex Ohmeda, Helsinki, Finland).

The interventional lung assist (ILA) (Novalung, Hechingen, Germany) was installed using the femoral blood vessels. One 17-Fr cannula was inserted into the femoral vein and two 13-Fr cannulae were inserted into both femoral arteries either via surgical preparation or via direct cannulation using Seldinger's technique with ultrasound guidance. Once the instrumentation was completed, 5,000 units heparin were administered. The ILA device was then filled with saline, connected with the cannulae and the extracorporeal circuit was established. The tubing for the O_2 _delivery into the ILA system was attached and the flow measurement through the arteriovenous shunt was initiated.

### Induction of acute lung injury

Acute lung injury was induced by bronchoalveolar lavage with 1.5 l warm saline, a modification of the method described by Lachmann and colleagues [21]. The lavage was repeated until the PaO_2 _was well below 100 Torr and remained stable for a period of 30 minutes at an FiO_2 _of 1.0. To maintain hemodynamic stability after the induction of acute lung injury, norepinephrine was continuously administered at 0.02–0.3 μg/kg per minute with an increasing dosage up to 0.1–1.8 μg/kg per minute by the end of the experiment. Basic volume therapy was initiated after induction of anesthesia using lactated Ringer solution. After induction of lung injury, when the blood pressure and heart rate indicated volume depletion, 6% hydroxyethyl starch solution was added.

### Protocol

The baseline data were collected after the completion of instrumentation before ILA was put into operation and lung injury was induced. The ventilator settings, the arterial, central venous, pulmonary artery and capillary wedge pressures, the cardiac output, the arterial and venous O_2 _pressures, the CO_2 _pressure and the respective hemoglobin concentrations and hemoglobin O_2 _saturations were determined.

The same data were collected after ILA was started before the initiation of lung lavage. Additionally, the O_2 _pressure, CO_2 _pressure, hemoglobin concentration and hemoglobin O_2 _saturation were determined in blood samples drawn from the outlet of ILA. Afterwards, the measurements were performed during the following three combinations of blood and gas flows through ILA: blood flow through one arterial cannula with no gas flow, blood flow through one arterial cannula with a gas flow of 2 lO_2_/minute, and blood flow through both arterial cannulae with a gas flow of 2lO_2_/minute.

Identical series of three measurements were repeatedly performed after the induction of severe lung injury. A total of three to four measuring series were acquired in each animal. Between the individual series, the extent of intrapulmonary arteriovenous shunting was varied by application of different positive end-expiratory pressures in the range 0–8 cmH_2_O and/or additional lavage. Data acquisition was started when the online PaO_2 _was stable. Care was taken to keep the conditions within each measuring series stable: no changes in ventilator settings or cardiocirculatory support were allowed until the data acquisition was completed.

After the completion of measurements, additional parameters such as the O_2 _content in arterial, mixed venous and ILA outlet blood, the O_2 _consumption and the O_2 _delivery through ILA were determined from the data acquired using basic physiological calculations. The intrapulmonary venous admixture (for instance, intrapulmonary right-to-left shunt) was calculated by the Fick equation.

### Statistical analysis

The results are presented as mean ± standard deviation values. Statistical analysis was performed using GraphPad Prism version 4.0 (GraphPad Software, San Diego, CA, USA). One-way analysis of variance followed by the Bonferroni multiple comparison test was applied to test the significance of differences between the measurements. The paired Student's *t *test was used to check the effect of the extracorporeal shunt flow on O_2 _delivery through ILA. Statistical significance was accepted at *P *< 0.05. The reported *P *values are two-tailed.

## Results

The results presented were obtained in seven animals during the following study periods: baseline conditions without ILA, ILA in operation before lung lavage, and ILA in operation after lung lavage. A total of 25 series of measurements were performed during the final period (for instance, after the induction of lung injury).

**Figure 1:**
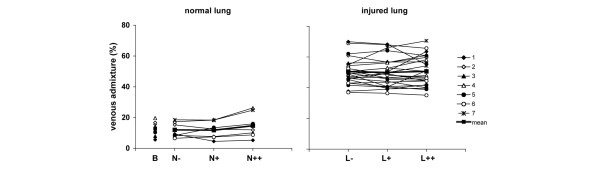
Venous admixture calculated in animals with normal and lavaged lungs. B, baseline; N-, normal lung, one arterial cannula open, no gas flow; N+, normal lung, one arterial cannula open, gas flow of 2 lO_2_/minute; N++, normal lung, two arterial cannulae open, gas flow of 2 lO_2_/minute; L-, lavaged lung, one arterial cannula open, no gas flow; L+, lavaged lung, one arterial cannula open, gas flow of 2 lO_2_/minute; L++, lavaged lung, two arterial cannulae open, gas flow of 2 lO_2_/minute.

In the present study, the effectiveness of ILA was followed under conditions of severe impairment of pulmonary gas exchange in a possibly large range of pulmonary arteriovenous shunting. During baseline conditions, in anesthetized and artificially ventilated animals, the pulmonary venous admixture was 12.5 (± 4.9)% (Figure 1, left). After ILA was put into operation the pulmonary venous admixture remained in the same range (Figure 1, left). The induction of acute lung injury by repeated bronchoalveolar lavage significantly raised the venous admixture to 50.5 (± 9.3)% (*P *< 0.001). During the subsequent measuring period, the pulmonary venous admixtures were in the range 35.0–70.6% (Figure 1, right).

Arterial systolic and diastolic blood pressures did not significantly differ among the measurements performed during baseline conditions, before and after lung lavage. Pulmonary capillary wedge pressures also remained unaffected: 8 (± 2) mmHg during baseline and before lavage, and 9 (± 3) mmHg after lavage. Cardiac output increased slightly but insignificantly after ILA was put into operation, from 6.8 (± 1.3) l/minute to 7.2 (± 1.8) l/minute, 7.7 (± 1.5) l/minute and 7.9 (± 1.5) l/minute under the three measuring conditions studied. After the induction of lung injury, cardiac outputs of 8.4 (± 2.6) l/minute, 8.7 (± 2.1) l/minute and 8.6 (± 2.3) l/minute were determined. These values did not significantly differ from those obtained before lavage.

**Figure 2:**
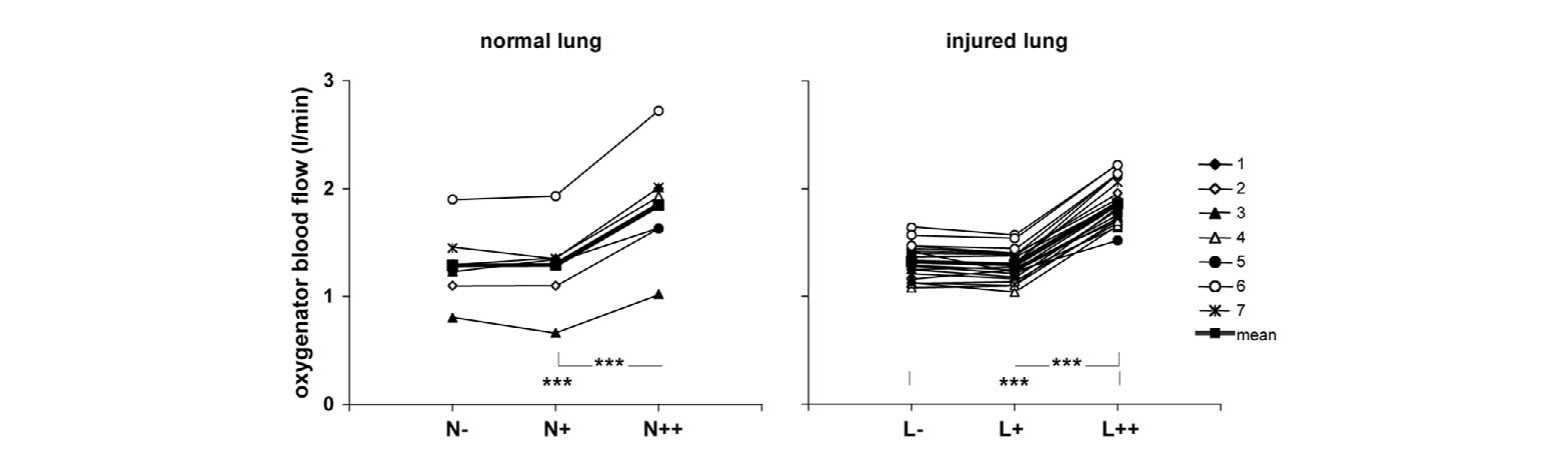
Oxygenator blood flow in animals with normal and lavaged lungs. B, baseline; N-, normal lung, one arterial cannula open, no gas flow; N+, normal lung, one arterial cannula open, gas flow of 2 lO_2_/minute; N++, normal lung, two arterial cannulae open, gas flow of 2 lO_2_/minute; L-, lavaged lung, one arterial cannula open, no gas flow; L+, lavaged lung, one arterial cannula open, gas flow of 2 lO_2_/minute; L++, lavaged lung, two arterial cannulae open, gas flow of 2 lO_2_/minute. ***P < 0.001.

The blood flow through the oxygenator was virtually independent of the lung condition and of the gas flow through the oxygenator (Figure 2). With one arterial cannula open and without gas flow, the blood flow through the oxygenator was 1.29 (± 0.37) l/minute before lung injury (Figure 2, left) and 1.32 (± 0.15) l/minute after bronchoalveolar lavage (Figure 2, right). After the addition of a sweep gas flow of 2 l O_2_/minute, the blood flow through the oxygenator remained unchanged at 1.29 (± 0.38) l/minute and 1.30 (± 0.14) l/minute, respectively. With two cannulae open and a sweep gas flow of 2 l O_2_/minute, the oxygenator flow increased to 1.85 (± 0.52) l/minute before lavage and to 1.86 (± 0.21) l/minute after the induction of lung injury (*P *< 0.001).

Before lung lavage and with one cannula open, the relative blood flow through the oxygenator corresponded to 18.5 (± 5.1)% and 17.0(± 4.5)% of the cardiac output during the measurements without and with gas flow through the oxygenator, respectively. The proportion of the oxygenator flow increased to 23.4 (± 5.2)% (*P *< 0.001) when both arterial cannulae were open. After the induction of lung injury, the corresponding relative blood flows through the ILA device were 16.6 (± 4.6)%, 15.5 (± 3.9)% and 21.7(± 4.9)% (*P *< 0.001) of the cardiac output, respectively.

**Figure 3:**
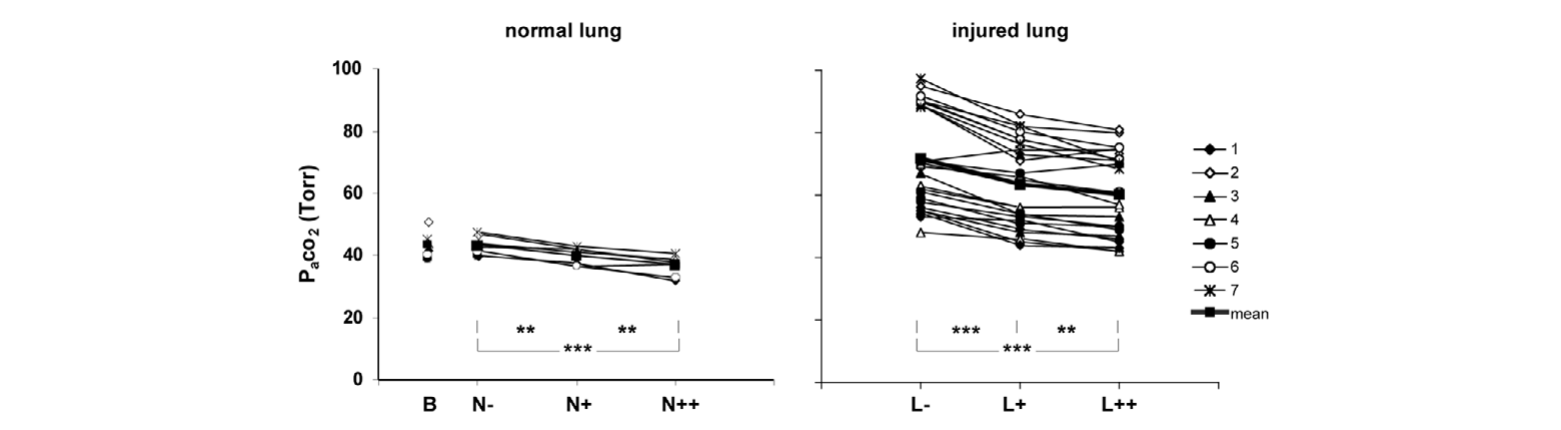
Arterial partial pressure of carbon dioxide (PaCO_2_) in animals with normal and lavaged lungs. B, baseline; N-, normal lung, one arterial cannula open, no gas flow; N+, normal lung, one arterial cannula open, gas flow of 2 lO_2_/minute; N++, normal lung, two arterial cannulae open, gas flow of 2 lO_2_/minute; L-, lavaged lung, one arterial cannula open, no gas flow; L+, lavaged lung, one arterial cannula open, gas flow of 2 lO_2_/minute; L++, lavaged lung, two arterial cannulae open, gas flow of 2 lO_2_/minute. **P < 0.01, ***P < 0.001.

Our measurements revealed a significant removal of CO_2 _by ILA both under the conditions of normal and injured lung (Figure 3). When compared with the baseline conditions, the operation of ILA with blood flow through one femoral artery and no sweep gas flow did not exhibit any effect on PaCO_2 _(44 (± 3) Torr versus 43(± 3) Torr). Both the addition of the gas flow of 2lO_2_/minute and the opening of the other femoral artery significantly decreased the PaCO_2 _values to 40(± 3) Torr (*P *< 0.01) and 37 (± 3) Torr (*P *< 0.001), respectively (Figure 3, left). During severe lung injury, the employment of ILA in the three settings studied led to a significant fall of PaCO_2 _from 72(± 17) Torr to 64(± 14) Torr (*P *< 0.001) and 60(± 13) Torr (*P *< 0.001), respectively (Figure 3, right). This was the result of the effective removal of CO_2 _by ILA as reflected by blood gas analysis performed on blood samples taken at the ILA outlet. The addition of the sweep gas flow reduced the pressure of CO_2 _from 71 (± 16) Torr to 31 (± 9) Torr (*P *< 0.001) during the ILA operation with one cannula open.

**Figure 4:**
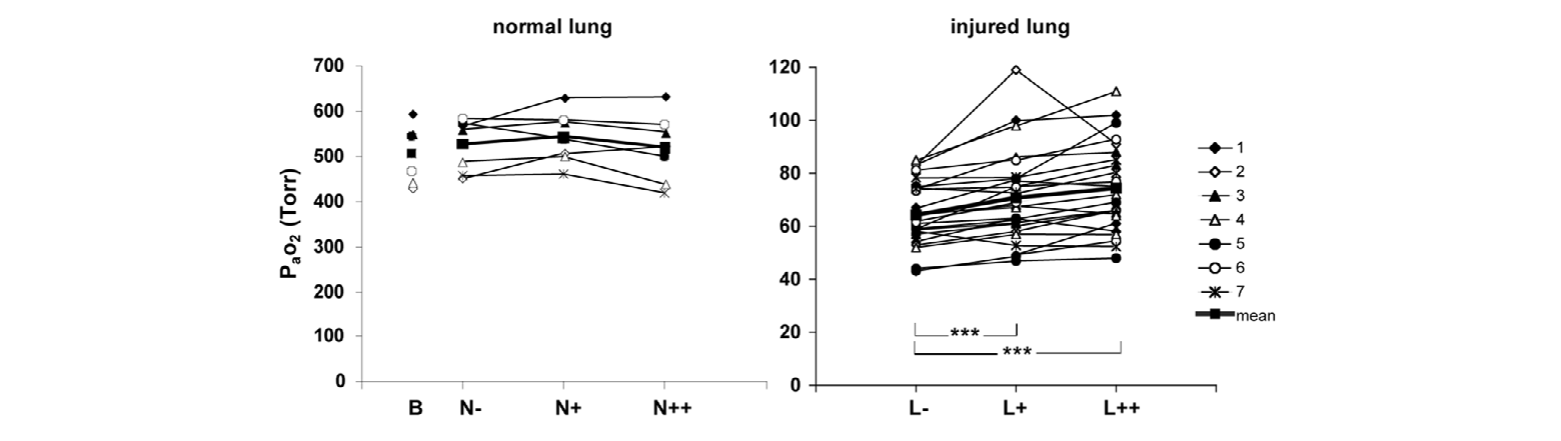
Arterial partial pressure of oxygen (PaO_2_) in animals with normal and lavaged lungs. B, baseline; N-, normal lung, one arterial cannula open, no gas flow; N+, normal lung, one arterial cannula open, gas flow of 2 lO_2_/minute; N++, normal lung, two arterial cannulae open, gas flow of 2 lO_2_/minute; L-, lavaged lung, one arterial cannula open, no gas flow; L+, lavaged lung, one arterial cannula open, gas flow of 2 lO_2_/minute; L++, lavaged lung, two arterial cannulae open, gas flow of 2 lO_2_/minute. ***P < 0.001.

No oxygenation effect of ILA was observed before lung lavage was initiated (Figure 4, left). The animals were ventilated at an FiO_2 _of 1.0, and consequently a high PaO_2 _value of 505 (± 62) Torr was found during the baseline conditions. During the operation of ILA with one cannula open without and with gas flow as well as with both cannulae open, the following PaO_2 _values were determined: 526 (± 58) Torr, 542 (± 58) Torr and 519 (± 75) Torr, respectively. After the induction of severe lung injury, a small but significant increase in PaO_2 _was observed when ILA was put into operation. Under the three ILA settings studied, the arterial oxygenation rose from 64 (± 13) Torr to 71 (± 14) Torr (*P *< 0.001) and 74(± 16) Torr (*P *< 0.001), respectively (Figure 4, right). The hemoglobin O_2 _saturation at the ILA outlet was 100% with the sweep gas flow turned on.

**Figure 5:**
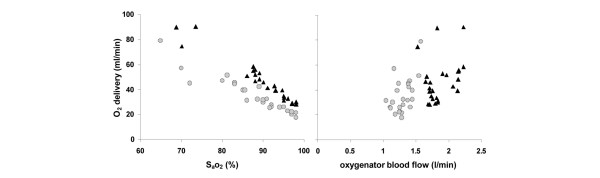
Oxygen delivery through ILA. One (circles) or two (triangles) arterial cannulae open.

The O_2 _delivery through ILA was significantly increased by the higher extracorporeal shunt flow during operation with both femoral arteries open when compared with the state when only one femoral artery was open (Figure 5). The corresponding mean volumes of O_2 _delivered were 47 (± 17) ml/minute and 36 (± 14) ml/minute, respectively. These amounts of O_2 _were equal to 16.8 (± 6.0)% and 12.5 (± 5.0)% of the total O_2 _consumption, respectively. The lower the hemoglobin O_2 _saturation in the arterial blood entering the oxygenator, the higher the amount of O_2 _added by ILA. The O_2 _delivery through ILA correlated linearly with the arterial hemoglobin O_2 _saturation and the oxygenator blood flow with the following equation: *y *= -1.66 *x*_1 _+ 24.88*x*_2 _+ 148.83 (*r*^2 ^= 0.94), where *y *is the volume of O_2 _(ml) delivered per minute, *x*_1 _is the hemoglobin O_2 _saturation and *x*_2 _is the oxygenator blood flow.

**Figure 6:**
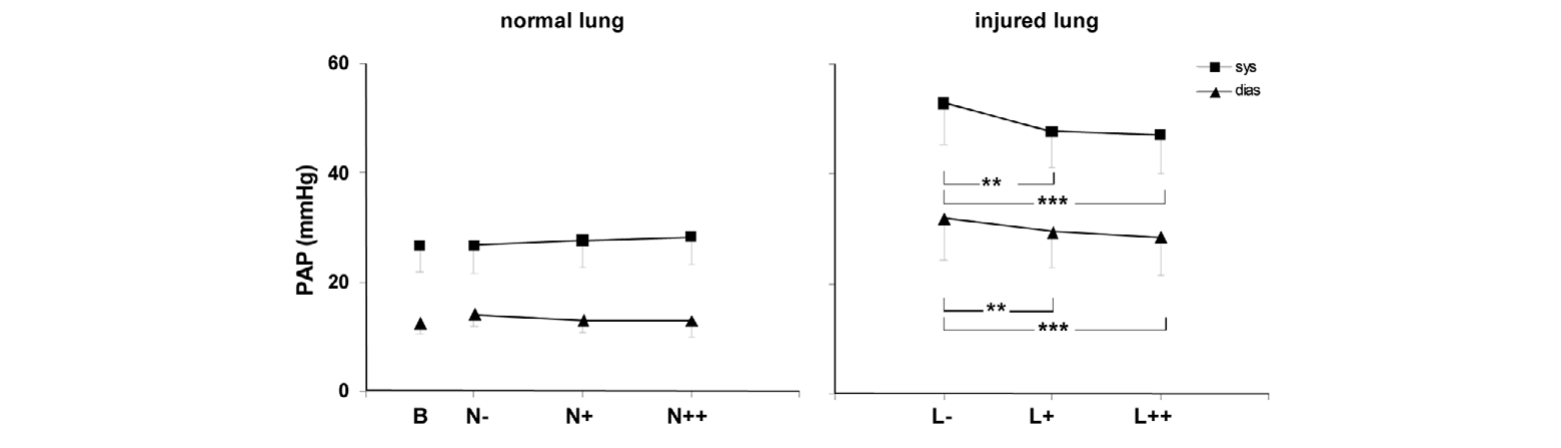
Pulmonary artery pressures (PAP) (mean ± standard deviation) in animals with normal and lavaged lungs. sys, systolic; dias, diastolic; B, baseline; N-, normal lung, one arterial cannula open, no gas flow; N+, normal lung, one arterial cannula open, gas flow of 2 lO_2_/minute; N++, normal lung, two arterial cannulae open, gas flow of 2 lO_2_/minute; L-, lavaged lung, one arterial cannula open, no gas flow; L+, lavaged lung, one arterial cannula open, gas flow of 2 lO_2_/minute; L++, lavaged lung, two arterial cannulae open, gas flow of 2 lO_2_/minute. **P < 0.01, ***P < 0.001.

Our study also revealed that both the systolic and diastolic pulmonary artery pressures, which were significantly elevated after the induction of acute lung injury, experienced a statistically highly significant fall during ILA operation with the 100% O_2 _gas flow of 2 l/minute when compared with ILA operation without this sweep gas flow (Figure 6). The systolic pulmonary artery pressure fell from 53 (± 12) mmHg to 48 (± 9) mmHg (*P *< 0.01) and 47 (± 10) mmHg (*P *< 0.001), and the diastolic pulmonary artery pressure from 32 (± 8) mmHg to 29 (± 7) mmHg (*P *< 0.01) and 29 (± 7) mmHg (*P *< 0.001), respectively.

## Discussion

The application of extracorporeal respiratory support in ARDS patients is currently recommended as a tool for minimizing the invasiveness of mechanical ventilation [22,23]. Less aggressive ventilator settings can be used in patients when extracorporeal membrane oxygenation is in operation because CO_2 _is removed not only by the lungs but also by the oxygenator. The effective CO_2 _removal has been well documented for the pumpless arteriovenous extracorporeal oxygenators [16-19,24,25] and has been confirmed in our study as well.

The primary aim of our experiments was to examine the oxygenation effect of the pumpless arteriovenous extracorporeal assist device. At present there exist only few studies in which the oxygenation effect of ILA was followed in a clinical setting. Reng and colleagues claimed to have 'relevant oxygenation' achieved in eight out of their 10 patients [26], Bein and colleagues demonstrated an improvement of oxygenation in 25 out of their 30 patients [27] and Liebold and colleagues found a significant improvement of the oxygenation index after 24 hours of treatment in a study of 20 ARDS patients [28]. Zimmermann and colleagues studied retrospectively data from eight patients with severe lung failure in whom ILA was applied during interhospital transportation. An effective removal of CO_2 _and a moderate increase in oxygenation was found [29]. The experimental studies aimed at studying the O_2 _delivery with the pumpless arteriovenous extracorporeal assist were partly performed in normal animals. For instance, Sussmane and colleagues determined that only 19.5% of total O_2 _consumption were provided by the extracorporeal lung assist at the lowest hemoglobin O_2 _saturation of 60% induced in normal lambs by ventilation with a gas mixture with an FiO_2 _of 0.1 [20].

Our intention was to study the ILA application under experimental conditions that closely resembled the clinical situation encountered in patients suffering from severe lung injury. We therefore performed measurements in an animal model of acute lung injury induced by bronchoalveolar lavage. This experimental model enabled us to follow the effectiveness of O_2 _delivery through ILA during different, acutely modified states of impaired pulmonary gas exchange as reflected by the broad range of pulmonary venous admixture detected. During the experiments we could also easily check the effect of different blood flow rates through ILA using either one or both femoral arteries for providing the inlet flow to the oxygenator.

Our findings correspond with the theoretical considerations regarding the O_2 _transport in blood. In general, the amount of O_2 _that can be added into the bloodstream is expected to depend mainly on the flow rate and the degree of desaturation of hemoglobin (as the amount of physically dissolved O_2 _is rather low). This means that the following two prerequisites must be fulfilled if oxygenation is intended to be achieved by ILA: a sufficient blood pressure gradient must exist between the inlet and outlet of ILA, and the pulmonary gas exchange must be severely compromised so that the arterial blood entering the systemic circulation exhibits a substantial decrease in hemoglobin O_2 _saturation.

During our experiments, the hemodynamic status of the animals was sufficient to provide high and stable blood flow rates through ILA even after the development of acute lung injury. Cardiac output was relatively high due to vasopressor support and adequate fluid supply. The relative flow rates equaled approximately 16% and 22% of the cardiac output during operation of ILA with either one or both femoral arteries, respectively. The first prerequisite for O_2 _transfer through ILA stated earlier was therefore achieved and adequate ILA flow was secured in spite of the small internal caliber of femoral arteries in pigs. The second prerequisite was also fulfilled because the acute lung damage induced by bronchoalveolar lavage compromised the pulmonary arterialization of blood. The mean hemoglobin O_2 _saturation in the inlet blood was 87.6(± 8.8)% and 89.6(± 8.0)% during ILA operation with one or both femoral arteries open. In spite of these conditions, the O_2 _delivery through ILA comprised only 12.5 (± 5.0)% and 16.8 (± 6.0)% of the total O_2 _consumption. How relevant this contribution is to the oxygenation might be judged differently. In our opinion, the use of ILA is not justified if the oxygenation effect is the major purpose of this therapy approach. As stated by Pesenti and Patroniti [22], however, no clear recommendations on optimum PaO_2 _and PaCO_2 _values in ARDS patients exist at present. In any case, the CO_2 _elimination by ILA with its additional small oxygenation effect facilitates lung-protective ventilator management.

Our experiments also showed the immediate effect of ILA operation with an O_2 _flow rate of 2 l/minute on pulmonary arterial pressures after the development of acute lung injury. The significant fall in pulmonary artery pressures, more pronounced at higher shunt flows through ILA, is suggestive of a decrease in hypoxic pulmonary vasoconstriction. In fact, the O_2 _content in the blood entering the lungs significantly rose from 50 (± 14) ml/l to 61 (± 12) ml/l and 68 (± 13) ml/l with ILA operating with the smaller and higher shunt flows, respectively. Our data do not allow conclusions to be drawn regarding the existence of this effect under the conditions of less acute lung damage with possibly different pathogenetic mechanisms. The benefit of this influence of extracorporeal oxygenation on pulmonary circulation cannot be judged unambiguously at present. On the one hand, the lower pulmonary arterial pressures may lead to diminished edema formation; on the other hand, the ventilation/perfusion matching may deteriorate. To clarify these aspects of ILA use, measurements using other experimental models of lung injury and studies on patients with ARDS will be needed.

## Conclusion

Our experimental study showed that pumpless arteriovenous extracorporeal membrane oxygenator slightly improved oxygenation in an animal model of severe acute lung injury. The volume of O_2 _delivered depended on the shunt flow rate and the degree of hemoglobin desaturation and was rather small, on average not exceeding 17% of the total O_2 _consumption. The operation of ILA significantly reduced pulmonary arterial pressures, but the consequences of this effect on regional pulmonary gas exchange remain to be determined in future studies.

## Key messages

• 	ILA slightly improves oxygenation in an animal model of severe acute lung injury. This effect is small, however, and the use of ILA may not be justified if oxygenation is the primary therapy goal.

• 	The amount of O_2 _transferred depends on the blood flow through ILA and on the degree of hemoglobin desaturation in arterial blood.

• 	CO_2 _elimination by ILA is pronounced, which makes ILA beneficial in the treatment of acute lung injury by facilitating lung-protective ventilation strategies.

• 	ILA operation with an established sweep gas flow reduces pulmonary artery blood pressures.

## Abbreviations

ARDS = acute respiratory distress syndrome; CO_2 _= carbon dioxide; FiO_2 _= inspired fraction of oxygen; ILA = interventional lung assist; O_2 _= oxygen; PaCO_2 _= arterial partial pressure of carbon dioxide; PaO_2 _= arterial partial pressure of oxygen.

## Competing interests

The authors declare that they have no competing interests.

## Authors' contributions

GZ participated in design of the study, carried out the study and drafted the manuscript. IF performed the analysis and interpretation of the data and revised the manuscript. DS carried out the study and participated in the analysis of data. GS participated in the analysis of data. SP carried out the study. EC participated in the design of the study. FW carried out the study. JS participated in design and coordination of the study. NW conceived of the study and participated in design of the study, analysis and interpretation of data and revision of the manuscript. All authors read and approved the final manuscript.
